# An Evaluation of Power Side-Channel Resistance for RNS Secure Logic

**DOI:** 10.3390/s22062242

**Published:** 2022-03-14

**Authors:** Ravikumar Selvam, Akhilesh Tyagi

**Affiliations:** Department of Electrical and Computer Engineering, Iowa State University, Ames, IA 50010, USA; tyagi@iastate.edu

**Keywords:** power analysis, residue number system, secret sharing, side-channel attack

## Abstract

In this paper, residue number system (RNS) based logic is proposed as a protection against power side-channel attacks. Every input to RNS logic is encrypted as a share of the original input in the residue domain through modulus values. Most existing countermeasures enhance side-channel privacy by making the power trace statistically indistinguishable. The proposed RNS logic provides cryptographic privacy that also offers side-channel resistance. It also offers side-channel privacy by mapping different input bit values into similar bit encodings for the shares. This property is also captured as a *symmetry* measure in the paper. This side-channel resistance of the RNS secure logic is evaluated analytically and empirically. An analytical metric is developed to capture the conditional probability of the input bit state given the residue state visible to the adversary, but derived from hidden cryptographic secrets. The transition probability, normalized variance, and Kullback–Leibler (KL) divergence serve as side-channel metrics. The results show that our RNS secure logic provides better resistance against high-order side-channel attacks both in terms of power distribution uniformity and success rates of machine learning (ML)-based power side-channel attacks. We performed SPICE simulations on Montgomery modular multiplication and Arithmetic-style modular multiplication using the FreePDK 45 nm Technology library. The simulation results show that the side-channel security metrics using KL divergence are 0.0204 for Montgomery and 0.0020 for the Arithmetic-style implementation. This means that Arithmetic-style implementation has better side-channel resistance than the Montgomery implementation. In addition, we evaluated the security of the AES encryption with RNS secure logic on a Spartan-6 FPGA Board. Experimental results show that the protected AES circuit offers 79% higher resistance compared to the unprotected AES circuit.

## 1. Introduction

Side-channel attacks (SCA) are hardware cryptanalytic techniques used to reveal a secret data value, such as a key embedded into an algorithm by exploiting the implementation vulnerabilities. If two different values for a key or a subkey result in different measurements of a physical attribute, such as power, timing, electromagnetic radiation, or even acoustics, the privacy is lost through this physical leakage.

We differentiate power analysis attacks into two broad classes. When a secret is revealed through a strong correlation between power samples and the secret data value, we consider it to be a loss of side-channel privacy. If the secret is encrypted with a cryptographic technique and is revealed through traditional cryptanalytic techniques, it is labeled as a violation of cryptographic privacy. Most of the known techniques target side-channel privacy. This paper targets both side-channel and cryptographic privacy. Residue number systems (RNS) allow one to create multiple shares of a secret. Each of these shares can be computed independently. The resulting shares can be combined into a single result. This is akin to the traditional multiparty computation. RNS enables one form of multiparty computation. Any homomorphic multiparty computation technique can be used within the context of this paper. Many hardware implementation optimizations of RNS systems exist, making it more suitable for this research.

### 1.1. Related Work

In [1], Kocher et al. reported the first side-channel attack and showed that the power consumption of the device is highly dependent on intermediate values of the cryptographic algorithm. Internet of Things (IoT) devices are especially vulnerable to side-channel attacks due to an adversary having physical possession of the devices at the edge. Zhao and Suh [2] mount a power side-channel attack on an FPGA. This makes even a cloud rack node vulnerable to power side-channels.

To prevent such attacks, it is essential to randomize or mask the intermediate values to decouple them from the device power consumption. This is widely done through the input encoding function of the side-channel countermeasure approach. The input encoding function transfers the data into encrypted shares, which ensures security in terms of cryptographic privacy and side-channel privacy. The cryptographic privacy refers to the difficulty of decoding encrypted data through traditional cryptanalysis techniques. Besides cryptographic privacy, the output shares of the encoding function exhibit another interesting property called side-channel privacy. Side-channel privacy is the observable difference in power side-channel leakage on the data transitions between “0” and “1.” The primary objective of creating shares of the input data is to mask/randomize the power consumption such that the side-channel observations of computations on the input value “0” and “1” are indistinguishable.

Several countermeasure techniques have been proposed to counteract side-channel attacks in [3,4]. Secret sharing schemes [5] form one of the most popular countermeasure approaches which has been developed in cryptography for multiparty computation and for sharing secrets. An adaptation of secret sharing schema for SCA splits each original bit into multiple uncorrelated shares in order to prevent the device side-channel leakage. The main idea behind secret sharing schemes is to split the input data into multiple shares. All the data shares are processed independently in parallel. The result of computation at the primary output end contains multiple resulting shares for each expected primary result. These resulting shares are combined at the output end to reconstruct the primary output. These techniques improve the resistance against power analysis attacks by providing uniformity and data independence in power consumption of individual shares.

For perhaps the best known secret sharing schema, Ishai et al. [6] developed a bit-level secret sharing technique by splitting each input bit into t+1 shares. For each input value *x*, *t* shares are derived from *t* random values $r_{x1}$, $r_{x2}$, $r_{x3}$, …, $r_{xt}$. (t+1)st share is computed as *x*⊕$r_{x1}$⊕$r_{x2}$⊕$r_{x3}$⊕ …⊕$r_{xt}$. Their adversary model is a *t*-probing adversary, which is a stronger adversary than a power side-channel adversary. A *t*-probing adversary can probe up to any *t* circuit nodes per cycle. A *t*-private circuit does not reveal any information about any bit *x*, even with a *t*-probing adversary. This provides both power side-channel privacy and limited (to *t* nodes probing) cryptographic privacy. Park et al. [7,8] showed several practical constructions of *t*-private gates that optimize its area, energy, and number of random bits.

Mangard et al. [9] discussed a security flaw in private circuits. They stated that glitches contributed significant power consumption and showed how such glitches weaken the security of private circuits. Later, in [10], Zachary et al. showed a practical power analysis attack using correlation enhanced collision attack. A secret sharing scheme similar to *t*-private circuits called Threshold implementation was proposed by Nikova et al. [11]. This secret sharing technique is based on multiparty computation and provably secure against differential power analysis (DPA) with fewer assumptions over hardware leakage. However, the threshold implementation techniques are still vulnerable to higher-order power analysis attacks described in [12].

Higher-order side-channel analysis (HO-SCA) is physical cryptanalysis that exploits the combined leakage through the power consumption of multiple individual shares. This analysis uses higher-order statistical moments to recover the secret value of a cryptographic algorithm [13]. Most of the existing countermeasures are still vulnerable to such higher-order power analysis attacks for two reasons. First, the leakage of intermediate values is distributed over shares, which is the primary SCA mitigation technique rather than masking the share values. Further, these shares utilize a linear function to reconstruct the original data. Hence, it is relatively easy for an adversary to model the leakage of the shared secret implementation. Second, if the shares are processed together with common Vdd and ground pins, the combined power consumption leads to leakage from such a susceptible implementation on actual intermediate values. Further, if the secure implementation is still in Boolean space, then the adversary can model the leakage with a hypothetical secret value, along with some additional mask bits to correlate with the target implementation leakage.

Logic design styles to make power consumption independent of data values with dual rail logic include Sense Amplifier Based Logic (SABL) [14,15], Wave Dynamic Differential Logic (WDDL) [16]. Similarly, there are other techniques such as asynchronous logic design [17], clock randomization [18], and power distribution design through decoupling unit [19] to hide the data-dependent leakage within the hardware. These design styles offer power side-channel privacy, but not cryptographic privacy. The data is in an open, non-encrypted form. The more robust countermeasure techniques, such as *t*-private scheme, provide both power side-channel privacy and limited cryptographic privacy. A cryptographic adversary needs to observe t+1 shares in order to decrypt original values. Out of practical considerations, the value of *t* cannot be very large. This opens up space for a secure design style that is both power side-channel private and cryptographically private within the design space for secure system implementations. Our proposed RNS secure logic fills this need.

The residue number system is a well studied number theory system, utilized in the field of computer arithmetic [20], and digital signal processing [21,22] applications to achieve performance upgrades through parallel computation.

### 1.2. Proposed Approach

In this paper, we discuss a new secure design style based on [23,24]. Our approach is to transform a bit in the Boolean domain into multiple encrypted shares derived from residues in a residue number system. These residue shares exhibit homomorphism for the bit-wise operations such as AND and XOR. Our proposed scheme is well suited for a multi-core platform, where an application can exploit parallelism in security-related applications. Each encrypted share can be processed in a separate core independently. In this work, we present three different secure design styles with varying characteristics based on adversarial complexity and resource overhead. There are many variations to the base schema for residue generation depending on the adversary model and the desired resource overhead. We explore this schema space to come up with three possible secure design styles with varying characteristics. We evaluate the resistance of RNS secure circuits against various side-channel adversary models. Further, we implement the RNS secure logic and report its power side-channel resistance through power uniformity based metrics and success rates of power side-channel attacks. The side-channel power analysis attacks typically deploy machine learning (ML) classifiers such as linear discriminant analysis (LDA), quadratic discriminant analysis (QDA), and naive Bayes (NB). RNS secure logic exhibits the lowest success rates for machine learning-based attacks compared to *t*-private logic.

The switching uniformity can be evaluated either analytically or through a distance metric such as KL divergence [25]. A natural conclusion seems to be that as switching gets more uniform or KL divergence of power distribution over various values for the secret reduces, the success rate for power side-channel attacks should go down. However, we have observed that even with an increase in KL divergence for power, the power side-channel success rate has gone down. We speculate that cryptographic privacy, even if not directly addressing power uniformity, thwarts power side-channel attacks. An interesting trade-off between power side-channel privacy and cryptographic privacy to minimize the success rate of a power side-channel adversary exists, which we explored and discussed in [26] (the cited paper is the conference version of current work with preliminary results which was published in IEEE 31st International Conference on VLSI Design and 17th International Conference on Embedded Systems, VLSID 2018).

Additionally, we develop an analytical metric for the RNS encoder to quantify the conditional probability of the input bit state given the residue state. We analyze the RNS secure circuit with respect to switching uniformity and propose some enhancement techniques to achieve better uniformity. Further, we investigate the implementation of RNS logic with public and private moduli. The side-channel resistance of these implementations is studied. We also evaluate the security of our implementation through real power traces using specialized side-channel board. The result confirms that it provides good security against higher-order power side-channel attacks.

### 1.3. Motivation

RNS logic supports distributed computation over multiple shares while simultaneously retaining cryptographic and side-channel privacy. It enhances side-channel security where the computation pertaining to a secret is performed by multiple devices or sensors. Sensor and IoT arrays to monitor or control an environment can benefit from the RNS logic. A computation C(S,x) involving a secret *S* and a parameter *x* can be performed on multiple (*k*) sensors or devices as $C_{i}\left(S_{i},x\right)$ with the share $S_{i}$ for 0<i≤k. This hardens the computation C against side-channel leakage.

### 1.4. Paper Organization

This paper is organized as follows. In Section 2, the basic principles of the RNS secure circuit are described. Section 3 discusses the resilience characteristics of proposed techniques with respect to switching uniformity and symmetry property. The adversary models and hybrid schemes for better side-channel resistance are discussed in Section 4. Section 5 presents the practical implementation of different circuits and their results. Finally, Section 6 summarizes and concludes the paper.

## 2. Basic Principles

In this section, some basic principles for our approach are discussed. Our proposed scheme maps from the message space to the residue code space. Message space consists of binary values (“0” or “1”) and corresponding bit-level operations/gates. Residue code space consists of residue values represented with *l*-bits. These residues use modulo operations such as modular addition and modular multiplication.

In message space, we use ⨁ and & to denote the logical addition (XOR) and multiplication (AND) operations over $Z_{2}$. Similarly, we denote + for addition and · for multiplication in residue space over $Z_{n}$. A *q* bit vector m= ($x_{1}$, $x_{2}$, $x_{3}$, …, $x_{q}$) denoted by $\bar{x}$ represents data in message space and its equivalent residue code is represented by ($X_{1,m}$, $X_{2,m}$, $X_{3,m}$, …, $X_{q,m}$) denoted as $\bar{X}$.

RNS secure logic is based on a combination of homomorphic encryption and residue number system. We use homomorphic encryption to create encrypted shares. The binary input values are transformed from message space to residue code space. Additionally, the homomorphism preserves the mathematical integrity of binary message space in the residual value space. An input encoding stage, which need not be on the chip implemented with the RNS secure logic shares, performs the binary message space to residue space conversion. Any computing host can perform this conversion and transmit the residue shares over any link including a network. The binary gates have equivalent modulo operations which are applied over the encrypted shares. Once the results in residue space are computed, they are decoded into the binary space. Once again, decoding need not occur in the secure chip. The residue shares can be transmitted back to a client over a link, where the decoding can be performed. We start by describing the construction of the RNS secret sharing scheme. Our approach comprises three stages, an *input encoder*, an *RNS circuit*, and an *output decoder*.

*Input encoder*: The homomorphic secret sharing scheme encodes the input message using a function called Input encoder (*Enc*). The encoder *Enc* maps each binary input *x* to an *l*-bit residue code denoted by $X_{m_{i}}$, where $m_{i}$ is the chosen modulus. Modulus choice has an important role in recovering the output back in binary value from residue code space which will be described in the output decoder function. The variable *l* defines the size of residue space. We first choose an *l*-bit random value $r_{x}$ and modulus $m_{i}$ from the relatively prime moduli set M = {$m_{1}$, $m_{2}$, $m_{3}$, …, $m_{n}$} and $m_{n}$ is equals to $2^{l}$− 1. The encoding function is modulo addition of random value $r_{x}$ with binary input *x* over $m_{i}$ and the mathematical representation is given in Equation (1).

(1) $$\begin{array}{c} X_{m_{i}}=\left(x\;+\;r_{x}\right)\;\mathrm{mod}\;\;m_{i} \end{array}$$

The security of the RNS secret shares fully depends on the random value of $r_{x}$ and modulus $m_{i}$. Note that without the random value $r_{x}$, the input binary bit *x* is exposed in the residue domain. The modulus $m_{i}$ is typically chosen per chip implementation, whereas the random values $r_{x}$ are assumed to be refreshed for every instantiation. They are generated by a statistically tested random number generator.

### Switching Uniformity

Note that the two main goals of a secure logic family are (1) uniform switching or power distribution so that it is not data dependent, and (2) remove any correlation between intermediate values. Note that the *t*-private logic achieves both these goals. Through an induction-based proof, the inductive hypothesis establishes that input encoder output has these properties. 1-prob(x) denotes the probability that node *x* state is 1. 1-prob(x) is a fairly good indicator of its switching probability: 2∗ 1-prob(x)∗(1− 1-prob(x)). Note that 1-prob$\left(r_{i}\right)$ of a random bit is 0.5, a random bit holds state 1 with probability 1/2. Additionally, note that when an input bit *x* with arbitrary 0≤ 1-prob(x)≤1 is exclusive-ORed with a random bit $r_{i}$, 1-prob$(x⊕r_{i})=0.5$. These two facts establish that all of the (t+1) shares output by an encoder have 1-prob equal to 0.5. The entropy of any two random bits $r_{i}$ and $r_{j}$ is 2 bits, since they are not correlated (distribution of states 00, 01, 10, 11 is uniform). By this token, the entropy of *t* random bits is *t*, establishing the other property. For the inductive hypothesis, consider the *t*-private gate for AND (&). The two incoming vectors $X=(x_{0},x_{1},\cdots ,x_{t})$ and $Y=(y_{0},y_{1},\cdots ,y_{t})$ have these two properties by inductive hypothesis. If each row of shift and add multiplication of *X* and *Y* forms a share, the 1-prob$\left(x_{i}y_{j}⊕x_{i}y_{k}\right)$ is 0.5 given that each share has 1-prob equal to 0.5. However, there is a correlation between rows reducing their entropy. In fact, all the shares of *X* are revealed within a row, along with one share of *Y*—$y_{i}$ thereby loses cryptographic privacy. By using an additional random bit per row, the entropy is restored to t+1. We aim to show similar analytical uniform switching for secure RNS logic style.

**Theorem** **1.** *The output of the input encoder (Enc) is uniformly distributed over modulus $m_{i}$ or the set *{0, 1, …,* $m_{i}-1$}.*

**Proof.**  Let P denote the plaintext in the binary space, X denote the encrypted share in the residue space. The residue space $M_{X}$ = {0, 1, …,$m_{i}-1$}. *X* = ${Enc}_{r_{x}}\left(x\right)$, where the random value $r_{x}$ is uniformly distributed over $M_{X}$.

$$P\left(R=r_{x}\right)=P\left(X=X\right)=\frac{1}{\alpha },$$

$$\;\;\;\;\;\;\;\forall \;r_{x}\;and\;X\;\epsilon \;M_{X}$$

where α = $|M_{X}|$. To prove this statement,

$$P\left(X=X|P=x\right)=\frac{P(X=X)\cdot P(P=x)}{P(P=x)}$$

$$=P\left(X=X\right)\;=\;P\left(R=r_{x}\right)$$

   □

Thus, the input encoder function maps the binary input without any bias on the residue code space. For a given message, the output of input encoder is equiprobable for the chosen modulus $m_{i}$. The same encoder function can be used to generate different shares by choosing different moduli $m_{i}$ with the same random value r${}_{x}$.

**Figure 1:**
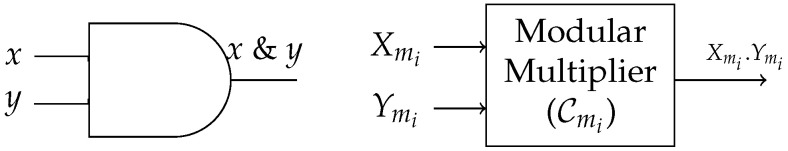
RNS circuit AND.

*RNS circuit*: Our goal is to transform the binary operators, such as AND and XOR, into equivalent residue operators using the composition of modulo multiplication and modulo addition in order to perform the operation securely. We constructed an RNS circuit that computes the residue space equivalent of a Boolean AND as shown in Figure 1. The size of this circuit is independent of the number of shares. It depends only on the modulus size (*l*).

Consider an AND gate in the binary space with inputs *x*, *y* and output *z*. i.e., z=x&y. In our model, the Boolean AND operation is performed with modular multiplication of $X_{m_{i}}$ and $Y_{m_{i}}$ over moduli $m_{i}$.

$$Z_{m_{i}}=X_{m_{i}}\cdot Y_{m_{i}}\left(\mathrm{mod}\;m_{i}\right)$$

The perfect privacy of our proposed scheme requires that the intermediate values or the output values be uniformly distributed with respect to the moduli $m_{i}$. This leads to uniform switching distribution as well with 1-prob of each of the output bits of a residue output equal to 0.5.

**Theorem** **2.** * [Uniformity] Let f be any modulo function over $m_{i}$ with inputs $X_{m_{i}}$, $Y_{m_{i}}$ and output $Z_{m_{i}}$. Then, the output $Z_{m_{i}}$ is uniformly distributed over residue code space, given that inputs are generated by an Input encoder (Enc)*.

*Output Decoder*: Each output share is computed independently for the given input vector for each modulus $m_{i}$. The output residue code *Z* is defined as linear congruence to the output of binary value *z* with respect to modulus $m_{i}$. To compute the resultant binary output bit, we apply Chinese remainder theorem (CRT) on the output shares obtained from the RNS circuit.

**Theorem** **3** (Chinese Remainder Theorem)**.** *Suppose that ℧⊂M, where all the elements are pairwise co-prime. let $Z_{m_{1}}$, $Z_{m_{2}}$, …, $Z_{m_{k}}$ be integers ϵ℧. Then the system of congruences, $z\equiv Z_{m_{i}}$ (mod $m_{i}$) for 1≤i≤k, has a unique solution modulo M = $m_{1}\times m_{2}\times \cdots \times m_{k}$, which is given by:*

$$z\equiv Z_{m_{1}}\cdot M_{1}\cdot M_{1}^{⋆}+Z_{m_{2}}\cdot M_{2}\cdot M_{2}^{⋆}+\ldots +Z_{m_{k}}\cdot M_{k}\cdot M_{k}^{⋆},$$

*where $M_{i}=M/m_{i}$ and $M_{i}^{⋆}\equiv {\left(M_{i}\right)}^{-1}$ (mod $m_{i}$) for 1≤i≤k.*

**Proof.**  Notice that gcd($M_{i}$,$m_{i}$)=1 for 1≤i≤k. Therefore, the $Z_{m_{i}}$’s all exist. Now, notice that since $M_{i}\cdot M_{i}^{⋆}\equiv 1$ (mod $m_{i}$), we have $Z_{m_{i}}\cdot M_{i}\cdot M_{i}^{⋆}\equiv Z_{m_{i}}$ (mod $m_{i}$) for 1≤i≤k. On the other hand, $Z_{m_{i}}\cdot M_{i}\cdot M_{i}^{⋆}\equiv 0$ (mod $m_{j}$) if j≠i. Thus, we see that $z\equiv Z_{m_{i}}$ (mod $m_{i}$) for 1≤i≤k. □

To apply Chinese remainder theorem, it is important that the modulus values $m_{i}$ used to create shares have to be relatively prime to each other. Further, in order to remove the mask, the value *e* has to be subtracted from the output of CRT followed by mod 2 operation. For this example, the value *e* is calculated as $r_{x}y+xr_{y}+r_{x}r_{y}$.

The RNS secret sharing scheme follows a variant of (k,t,n)-threshold scheme [27]. Our threshold scheme is defined in Definition 1. The RNS secret sharing scheme requires a minimum of 2 shares to decode the result residue shares to binary output. Additionally, the shares chosen for decoding must be computed with moduli that are co-prime.

**Definition** **1.** ***(2,k,n) threshold secret sharing scheme:*** *Let n be an integer, n≥3, and 3≤k≤n. A (2,k,n)-threshold secret sharing scheme is a method for generating shares for x as P = {$X_{m_{1}}$,$X_{m_{2}}$, …$X_{m_{n}}$} such that*

- *For any A⊂P such that |A|<2, learning the element x should be difficult.*
- *For any A⊂P such that |A|=2, reconstruction of element x is possible, given that $gcd(m_{i},m_{j})=1$.*
- *For any A⊂P such that |A|≥k, reconstruction of the element x becomes easier, given the set $\left\{X_{m_{i}}|i\epsilon A\right\}$ are relatively prime.*

## 3. RNS Logic Resilience Characteristics

In this section, we discuss the resilience characteristics of our proposed scheme. We first review the more general definition of the masking technique and then we will show how our proposed approach is resilient to side-channel attacks.

**Definition** **2.** ***Masking:*** *An intermediate value v masked with r results in a masked value $v_{r}$ = f(v,r) which is independent of v. The intermediate value is said to be masked, if the power consumption of $v_{r}$ is independent of v.*

In general, there are known techniques and frameworks for side-channel attacks. An adversary identifies the vulnerable point of an encryption algorithm which is denoted as the targeted intermediate value. The intermediate values are typically computed from the targeted secret and some other input under adversary control. The targeted intermediate value should have high controllability for the adversary to perform successful practical attack. The adversary develops an a priori relationship model between the secret and the targeted intermediate value. This model allows an adversary to distinguish the secret value based on the side-channel leakage from the intermediate value.

### 3.1. Symmetry Property

In an RNS secure logic, the encoding scheme converts all the binary inputs to residue space using Equation (1). The random value $r_{x}$ masks the binary value by applying the modulo addition operation. Unlike the other side-channel countermeasures, the shares are created by modular addition. This has the potential to reveal the relationship between a residue and the corresponding input bit through the distributions of bits within the residue. Over the space of all the hidden parameters, modulus $m_{i}$, and the random value $r_{x}$, an input bit 0 maps to many residues—set $R_{0}$. Similarly, input bit 1 maps to a set of residues $R_{1}$. Ideally, the two sets $R_{0}$ and $R_{1}$ should not be distinguishable to the adversary. This property called *residue indistinguishability* or *symmetry* hides the input to residue relationship. The sample RNS residue encoding is shown in Table 1 for 2-bit residue space over all possible $r_{x}$ and moduli $m_{i}$. The valid moduli set for 2-bit encoding is {2, 3}.

The columns $X_{m_{1}}$ and $X_{m_{2}}$ are the outputs of the RNS encoding computed with the modulus values 2 and 3, respectively. Based on input binary values, the residue output shares are organized into two sets, one for binary “0” and another for binary “1”. In Table 1, The blue circle shows the share values for binary “0” and the red circle shows the share values for binary “1”. The $X_{m_{1}}$∪$X_{m_{2}}$ contains {00, 01, 10} for *x*=0. Similarly for *x*=1, the $X_{m_{1}}$∪$X_{m_{2}}$ contains {00, 01, 10}. The residue sets for x=0 and x=1 contain the same residue values. Given the residual share values, it is difficult for an adversary to infer the binary input value without knowing the random secret $r_{x}$ and moduli $m_{i}$.

**Figure 2:**
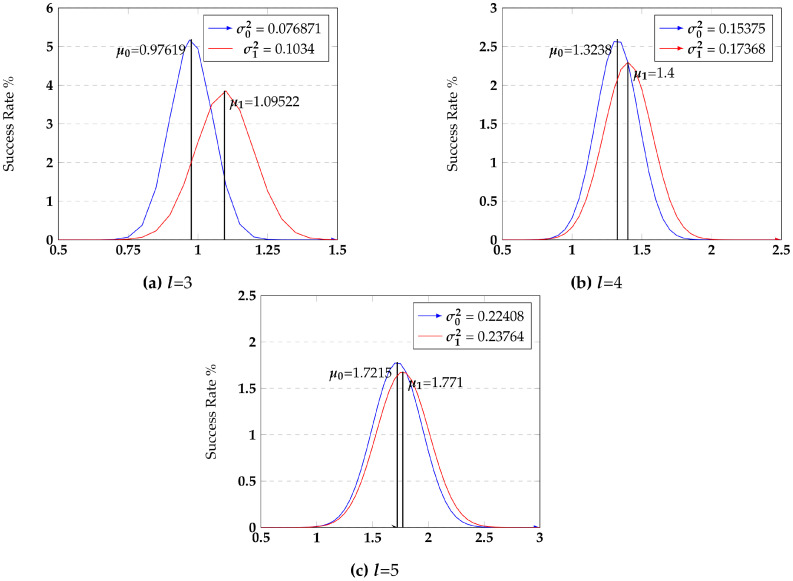
Distributions of residue shares.

We extend this observation into a quantitative measure called *symmetry*. Symmetry is the probability that adversary fails to distinguish the input bit state given the residue value distribution. In a realistic attack, the adversary does not have access to the residue values. It infers a residue state through a power side-channel. Traditionally, these power models are Hamming-weight-driven. Hence, the primary differentiating characteristic between different residues is their Hamming weight difference. The adversary attempts to gain incremental information about the input bits state 0 or 1 by measuring infinitesimal differences in the average Hamming weight associated with the residues of input bit 0 and 1. If these average Hamming weights are identical, perfect symmetry exists, denying the adversary this information. Equation (2) models this intuition for a fixed modulus value $m_{i}$. The targeted chip is functional with a fixed $m_{i}$, and hence the uncertainty/averaging space for the adversary comes from the random mask $r_{x}$. The average Hamming weight distributions are plotted in Figure 2 for various values of residue size.

(2) $$S{\left(x\right)}_{m_{i}}=\sum_{r=0}^{2^{l}-1}\frac{HW(\left(x+r\right)mod\;m_{i})}{2^{l}}$$

where *i* varies from 1 to ⌈M⌉. *r* is a random value.

As reported in [25], we use KL divergence SCA metric to study the symmetry of residue shares with respect to binary values. We computed the KL divergence metric to find the distance between the two distributions for input bits 0 and 1 for all l=3, 4, 5. Smaller KL divergence values indicate that the 0 and 1 distributions are close to each other, and hence less differentiable and more symmetric. Table 2 reports both KL divergence and the symmetry values over the residue space sizes.

Machine learning offers a powerful model-building technique to an adversary to correlate the Hamming weight of the residue reflected in the measured power trace and the input bit state. We assess the machine learning classifiers to validate the symmetry metric/property to demonstrate that higher symmetry results in lower correlation. These results are reported in Table 2. The success rate column should be interpreted within the context of a random decision. Since the decision in this context is guessing the input bit state for a given residue Hamming weight, a random coin toss has success probability of 0.5. Any higher success probability indicates machine learning’s advantage. The key thing to note is that as symmetry increases, the success rate of ML gets closer to a random guess. Note that we gave an advantage to the ML classifier by having it guess the input bit state from the actual residue state rather than the Hamming weight of the residue. It is evident that the RNS encoding scheme provides strong cryptographic privacy to mitigate the power side-channel attack.

### 3.2. Symmetry in a Software Implementation of RNS

Our proposed encoding scheme is based on homomorphic encryption, which can be used to provide security to cloud-based applications as well. Recall that residue sizes were limited to a small number, such as five, by practical circuit implementation constraints. In software, however, the residue sizes *l* can be scaled to a large number. We extended our *symmetry* analysis to software implementations with larger values for *l*. Due to processor implementation characteristics, *l* would need to be a multiple of byte size. We experimented with *l* equal to 16 and 32 bits (2 and 4 bytes). This gave us an asymptotic view of *symmetry* metric effectiveness.

**Figure 3:**
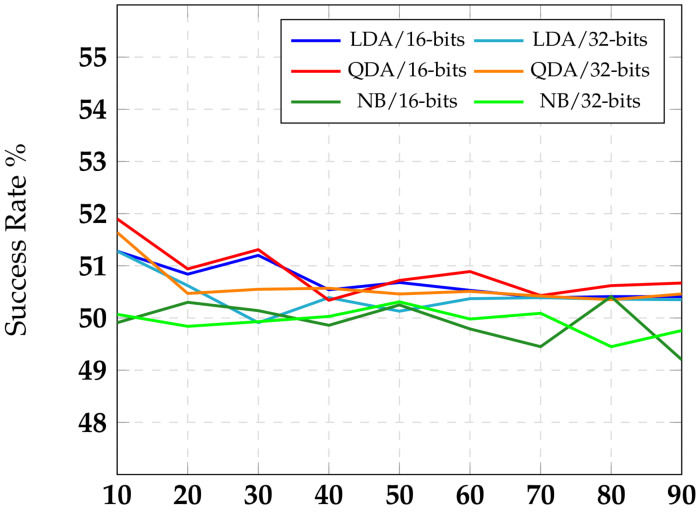
Success rate of the RNS encoding.

Computing the symmetry values for l=16, 32 is tedious and requires $2^{l}$ iterations based on Equation (3). For l=5, symmetry value is already at 0.99. With higher *l* values, it would converge towards 1 with error converging to 0. Hence, we did not compute the actual symmetry values. We, however, applied the three machine learning classifiers, (LDA, QDA, and NB), to predict input binary values from the residue share values. The *x*-axis in Figure 3 denotes *ratio*, which is the ratio of training dataset size to the test dataset size. Note that a higher ratio should make machine inference converge to a truer success rate. The *y*-axis captures the success rate of ML classifier. Once again, the ML classifier has an advantage only if its success rate is better than the random 50%. We expect symmetry to be better with l=32 than with l=16. It is reflected in Figure 3 with a tighter band around 50% success rate line for l=32 classifier results compared to the l=16 classifier results. The differences between the classifiers have to do with their native characteristics.

**Figure 4:**
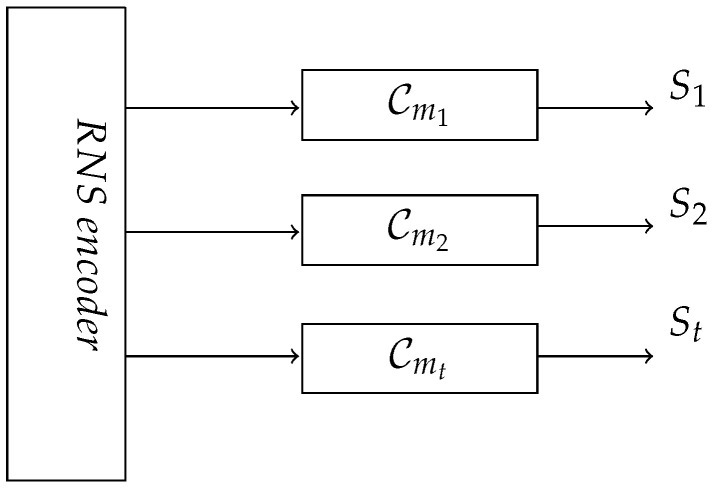
RNS secure circuit’s multi-lane computation.

It is clear that the adversary will not gain any advantage even with model-based attacks. Over the unknowns r,m, let $f_{X}^{0}=\left|Enc\left(0,r,m\right)=X\right|$ for $0\leq r\leq 2^{l}-1$ and $2\leq m\leq 2^{l}-1$. $f_{X}^{0}$ gives the frequency with which a 0 is encoded in to the residue *X* for uniformly distributed unknowns r,m. We can similarly define $f_{X}^{1}$, the frequency with which a 1 is encoded into the residue *X*. Ideally $f_{X}^{0}=f_{X}^{1}$ for all *X*. A weaker symmetry allows $f_{X}^{0}\neq f_{X}^{1}$, but then insists on $f_{X}^{0}=f_{X}^{\prime 0}$ and $f_{X}^{1}=f_{X}^{\prime 1}$ for all residues *X* and $X^{\prime }$. However, in reality, often $\exists X,X^{\prime }$ such that $f_{X}^{0}\neq f_{X}^{\prime 0}$ or $f_{X}^{1}\neq f_{X}^{\prime 1}$. These differences create a skew in the transition probability of residue bits, potentially targetable by an adversary. We investigate the transition probability distribution and discuss in Figure 4 a technique to achieve more balanced transition probability for RNS secure circuits.

### 3.3. Multi-Lane Computation

In addition to the residue indistinguishability property, our proposed scheme has an interesting characteristic called multi-lane computation. The RNS encoding function creates encrypted shares with respect to the moduli $m_{i}$. These share values are congruent with each other, which allows the hardware designer to implement separate hardware for each share. This is a unique characteristic of RNS secure circuit.

In ideal scenarios, the secret sharing scheme splits the input data at the primary inputs end and combines the primary output shares at the end of computation. For side-channel countermeasures, a secret sharing scheme such as *t*-private circuits divides the input data into t+1 shares. Each original gate is replaced by special gates capable of handling t+1 shares for each input. An input *x* is encoded with *t* random shares $x_{0},x_{1},\cdots ,x_{t-1}$ with $x_{t}=x_{0}⊕x_{1}⊕\cdots ⊕x_{t-1}⊕x$. For intermediate values, the t+1 shares are created by the intermediate gate design. Use of a random bit to create shares randomizes the transition probability of the bit to 0.5 for both transitions minimizing the power based information leakage. In *t*-private logic, when intermediate shares that are correlated (as in an & gate) are created, an additional random bit is used to restore this balanced transition property. In another logic family, threshold implementation, the intermediate bits of an & gate are handled through balancing of terms. The relevant aspect of these secret sharing scheme circuits is that the shares of a bit are entangled or not separable at each gate.

In contrast, for RNS secure circuits, the share values support homomorphic computation on each share. We convert a Boolean circuit into its equivalent RNS circuit where the computation with respect to the shares derived from modulus $m_{i}$ can proceed in its own computation lane. This gives rise to *t* independent computation lanes, which need only be combined at the output stage at the end of computation lane. Each share can be processed with its own hardware lane as shown in Figure 4. The RNS secure circuits for *t*-shares are denoted as $C_{m_{1}}$, $C_{m_{2}}$, …, and $C_{m_{t}}$. The power consumption data captured from each RNS lane are denoted as $P_{m_{1}}$, $P_{m_{2}}$, …, and $P_{m_{t}}$, respectively. The *i*th RNS lane takes the input $X_{i}$ and $Y_{i}$, and generates the residue output $S_{i}$.

In this setup, the power side-channel adversary attacks each share independently which we call *singular attack mode*. For this attack mode, we assume that the adversary controls some of the binary inputs to the circuit before the lane shares are created in an encoder and observes the power consumption for all the relevant lanes. In power analysis, higher order attacks (HOA) have been shown to be more powerful. A corresponding attack could use the correlations between multiple lanes to extract the binary input to residue shares mapping. There are no existing methods on combining the leakage data of different share computations. Since we do not have a good mathematical model, lane correlation-based attacks are difficult to perform against RNS secure circuit. In singular attack mode, we partition the Lane *i* binary primary inputs into $<X_{i},Y_{i}>$, where the inputs $<X_{i}>$ are controllable by the adversary, but $<Y_{i}>$ are private. We assume that there are $b_{X}$ binary input bits in the set $<X_{i}>$ and $b_{Y}$ binary input bits in the set $<Y_{i}>$ with $N=b_{X}+b_{Y}$. The adversary’s goal is to retrieve the binary secret using the power leakage $P_{m_{i}}$ that is captured during the execution of a given function on input data $<X_{i},Y_{i}>$ in the *i*th lane. The power leakage of the RNS secure circuit computing a function *f* in Lane *i* is given in Equation (3).

(3) $$P_{m_{i}}=L_{Y_{i}}\left(f\right)+\bar{L_{\;Y_{i}}\left(f\right)}+\epsilon$$

where, $L_{Y_{i}}\left(f\right)$ is power leakage due to sensitive variable.

$\bar{L_{Y_{i}}\left(f\right)}$ is power leakage due to non-sensitive variable.

ϵ is Lane *i* device noise.

For a successful attack, the $L_{Y_{i}}\left(f\right)$ needs to be significant, which means that there should be a distinct difference in the probability density function for $P(L|Y_{i}=0)$ and $P(L|Y_{i}=1)$. However, the symmetry property of the RNS circuit ensures that the distance between the probability density function is minimal. Recall that each of the *N* input bits in $<X_{i},Y_{i}>$ results in an *l*-bit residue, which is further exclusive-ORed with an *l*-bit random word within the encoder. Hence, the unknown search space consists of N∗l random bits that are statistically not correlated. This increases the unknown search space for a key hypotheses to $2^{N*l}$. RNS secure circuits with different hardware lanes allow the designer to operate each lane at different clock frequency which affects the temporal alignment of the leakages from each lane.

For probing side-channel, the adversary has to probe all the shares of the same value in each lane at the same time. This requires large amount of resources with high precision, which is currently impractical. Even if the adversary is able to extract the residue shares, inferring the corresponding binary input *x* is still difficult without knowing the hidden parameters, random value $r_{x}$, and modulus $m_{i}$, as we have discussed before. In a multi-core device, each encrypted share could be processed independently on separate cores with a staggered unpredictable schedule. Moreover, power pins associated with different cores are isolated. The adversary will have to observe and capture the leakages of each share/lane/core separately.

## 4. Power Side-Channel Adversary

In this section, we will discuss the strength of the RNS secret sharing scheme and introduce hybrid schemes to achieve better resistance against any power side-channel attacks. We first define our basic assumptions for SCA target circuit. We assume that the adversary can control only binary input values. The encrypted shares are not exposed to an adversary which is in line with commonly accepted adversary models for a countermeasure technique. A power analysis attack is a type of a side-channel attack that exploits the leakage obtained in the form of power consumption from the target circuit. Masking techniques are used to randomize power consumption to make sure that the measured leakage is independent of any processed data. RNS secret sharing scheme is also a type of a masking scheme, which uses homomorphic encryption to mask the intermediate values. RNS secure circuits are highly resistant to power analysis because of their resilience characteristics defined in Section 3. More formally, we could describe the strength of RNS secret sharing scheme as follows.

The Definition 3 says that the adversary can successfully model the leakage, to distinguish the intermediate values between 0 and 1. This could be achieved only if the input value strongly determines the intermediate value.

**Definition** **3.** * [1] Let 𝒞 be a circuit under investigation with secret values $\dot{y}$. The differential power analysis is defined by*

$${▵}_{y,\dot{y}}\left(N,j\right)=\frac{\sum_{i=1}^{N}D\left(X_{i},y_{j}\right)C_{\dot{y}}\left(X_{i}\right)}{\sum_{i=1}^{N}D\left(X_{i},y_{j}\right)}-\frac{\sum_{i=1}^{N}\left(1-D\left(X_{i},y_{j}\right)\right)C_{\dot{y}}\left(X_{i}\right)}{\sum_{i=1}^{N}\left(1-D\left(X_{i},y_{j}\right)\right)}$$

*where 𝒟 is a function for the key hypotheses.*

Our RNS secret sharing scheme applies homomorphic encryption to the input values using the random value $r_{x}$ and the moduli $m_{i}$. Our encoding scheme completely weakens the control of input binary values over intermediate values by creating encrypted shares. Hence, the power analysis adversary is unable to model the leakage $D(X_{i},y_{j})$ for a successful attack. Additionally, the residue indistinguishability characteristics of the RNS secure circuit more or less equalize the power consumption values $C_{\dot{y}}\left(X_{i}\right)$ between all the transitions. Hence, the adversary is not able to distinguish the leakages with respect to the output binary level transitions. We believe that the cryptographic privacy of our proposed scheme also makes it difficult to distinguish based on power leakage.

**Figure 5:**
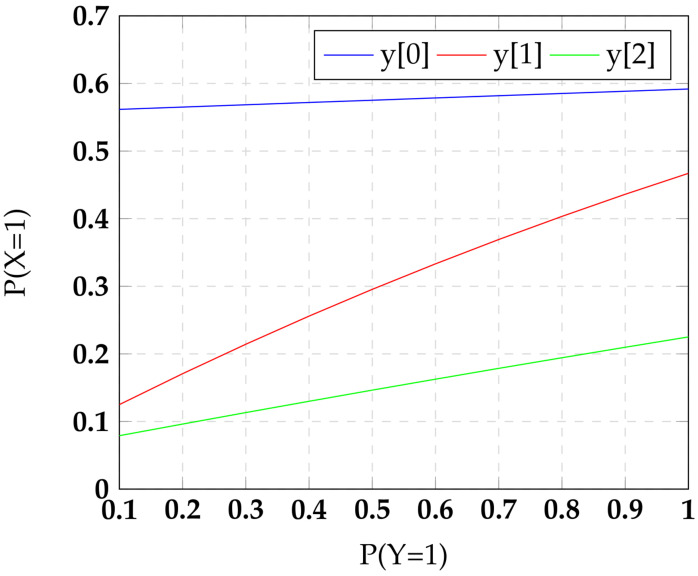
Transition probability of RNS encoding scheme.

In order to study the power leakage characteristic of our RNS circuit, we computed the switching probability for each output bit of RNS encoding scheme, as defined in Equation (1) with l=3. The input signal probabilities were propagated in a gate level description of an encoding scheme, and the results are plotted in Figure 5. The input values are single bit binary values which are exclusive-ORed with a least significant bit of a random value. The carry chain of this computation is designed using a logical AND gate and propagated to the following bits of random values to the most significant bit. The modular function truncates the overflow with respect to a chosen modulus value. This perturbs the uniformity of our scheme. The result shows that the output transition probability of our encoding scheme is skewed with the input signal probability. We have found that the modulus reduction reduces the effect of random value $r_{x}$ and makes the transition probability biased. Hence, it is more likely to be vulnerable to power analysis attacks with larger circuits.

**Figure 6:**
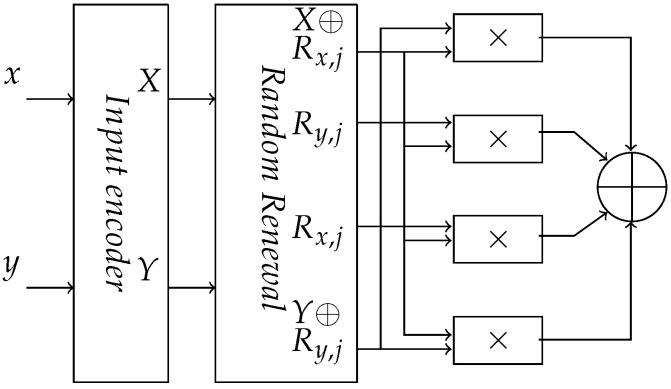
Secure RNS circuit with random renewal scheme.

To make the transition probability of the RNS circuit unbiased, we introduced a random renewal scheme as in an AND gate of *t*-private logic. In a random renewal scheme, we performed bitwise exclusive-OR function between a random value $R_{i,j}$ and the output of the encoder. The variables *i* and *j* refer to the input and the circuit stage, respectively. The random value $R_{x,j}$ and $R_{y,j}$ is *l*-bits wide with each bit distributed independently and uniformly. This makes the output transition probability of an RNS encoder 0.5 and unbiased. The modified secure RNS circuit is shown in Figure 6. The random renewal exclusive-OR operation maintains homomorphism over the residue values only with true multiplication. Therefore, no modulus reduction is performed. Once the recovery exclusive-OR operation has been done, the residue values are obtained by modulo reduction with appropriate modulus value $m_{i}$. To maintain the unbiased transition probabilities, random renewal techniques should be applied at each stage with independent random values.

**Figure 7:**
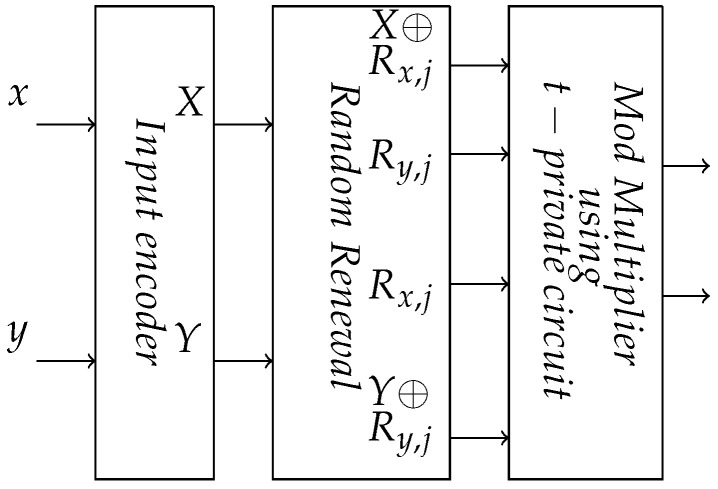
Secure RNS circuit with *t*-private circuit.

A hybrid logic family that merges *t*-private circuits with RNS circuits could have additional advantages. In this hybrid logic, the RNS shares are still created in the usual manner. However, the residue output bits are further encoded for *t*-private logic. For 2-hybrid logic, each share bit is split into two additional bit shares in the usual 2-private scheme—*x*, $x⊕R_{x}$. We used *t*-private logic gates to implement the equivalent RNS secure circuits, as shown in Figure 7. The *t*-private logic gate uses the secret random values to maintain the uniform switching property throughout the design. This technique provides higher security against side-channel attacks both in terms of secret search space and randomization of side-channel leakage—as we show experimentally in Section 5.

## 5. Results

We have implemented an RNS circuit for a boolean AND gate with l=3 using the 45 nm FreePDK Standard Cell library and Cadence analogue simulator (Spectre). We have conducted exhaustive simulations over residue space using ocean script. We have measured peak current and power consumption of all the possible input transitions $\left(2^{12}\right)$. We performed two styles of analysis of the simulated data: one uses a single random value for encoding the input variables, and the other one uses different random values for encoding the different input variables. First, we computed the average values for each class of output transition with respect to the binary values. Then, we have calculated the coefficient of variation and Kullback–Leibler divergence for each logic scheme shown in Table 3.

In addition to the base RNS scheme, we have evaluated the SCA metric for random renewal techniques with separate, per-share random variables. The hybrid scheme that includes *t*-private logic also uses separate, per-share random variable in the encoder. With increased randomness due to the random variables in the *t*-private logic and additional random variables per encoder, we expected to see lower standard deviation and KL divergence. Intuitively, increased randomness resulted in increased uniformity in the switching distribution.

**Figure 8:**
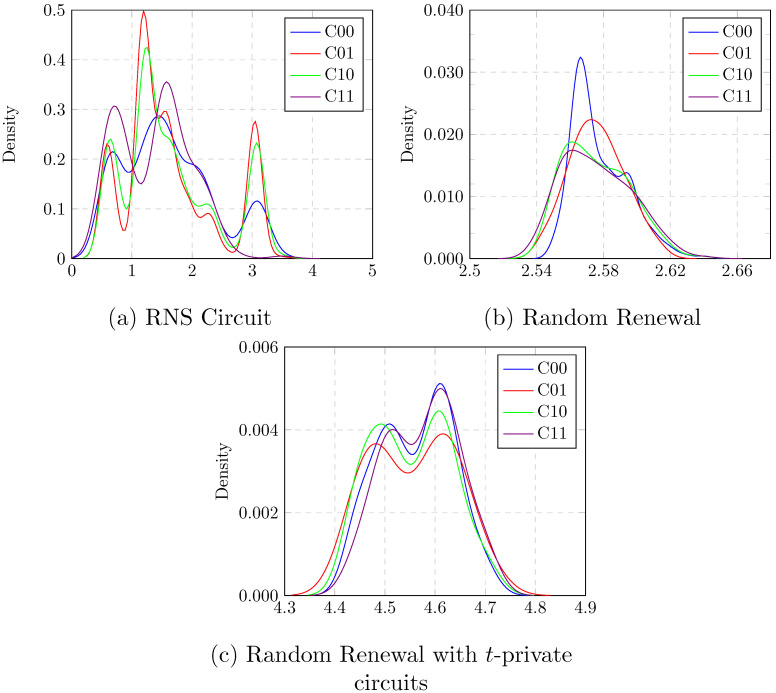
Probability density of maximum current.

The coefficient of variation is a well known SCA metric used to quantify the effectiveness of the countermeasures. The lower the value, better the resistance against power analysis attacks. In our scheme, the coefficient of variation is likely to converge towards lower values for larger circuits. The probability density function of peak current was calculated for each output transition with respect to the binary values and the results are plotted in Figure 8.

Additionally, we computed another SCA metric using Kullback–Leibler (KL) divergence for our analysis which defines the failure probability of the attack. We compute the KL divergence between all pairs of transitions and find the maximum values to identify the transition pair with higher deviation. KL divergence is a measure of how far apart, and hence how distinguishable, two probability distributions are. Table 3 indicates that base RNS scheme is the least SCA-resistant, followed by random renewal, followed by random renewal with *t*-private as most resistant. Even when we use multiple, separate random values per-share, the relative SCA-resistance follows the same order: base RNS < random renewal < random renewal with *t*-private. Furthermore, observing Table 3 and Table 4 together, each of the schemes (1) base RNS, (2) random renewal and (3) random renewal with *t*-private, shows higher resistance with a per-share random value instead of a single shared random value. The total SCA-resistance order among these six schemes appears to be base RNS–single random < random renewal–single share < base RNS–multiple random < random renewal with *t*-private–single random < random renewal–multiple random < random renewal with *t*-private–multiple random.

As shown in Table 4, the KL divergence SCA metric value is 0.1620 for the random renewal-multiple random scheme, which corresponds to about an 80% failure probability [25], leading to an expected machine learning success rate of 20%. Similarly, the KL divergence SCA metric is 0.0688 for random renewal with the *t*-private-multiple random scheme. This corresponds to a failure probability of 90%.

In order to validate the KL divergence driven metric and its anticipated failure probability, we also applied machine learning based classification such as linear discriminant analysis (LDA), quadratic discriminant analysis (QDA), and naive Bayes (NB). We have recorded the peak current for RNS secure circuit and its variants for 5000 randomly generated inputs. For each classifier, we classified the measured leakage data into a training set and validation set with a ratio of 4:1. The success rate was then computed for each classifier and the results are given in Table 5. The random renewal schemes are more resistant than either of the base *t*-private or base RNS schemes. Generally, the SCA resistance order base *t*-private < base RNS < random renewal < random renewal with *t*-private is maintained for all the classifiers with a few exceptions. In *t*-private logic, each AND and OR gate, requires additional *t* random variables. This however, would complicate SPICE simulations significantly. Hence, we ended up using weaker versions of *t*-private logic where all AND gates share the same single random variable and so do all OR gates. This explains some of the unexpected results in Table 5.

### 5.1. Modular Multiplication

For RNS logic, a basis for arithmetic functions could be addition and multiplication. These adders and multipliers operate on *l*-bit values. Overflow can occur both at X+Y and X∗Y. Modular reduction results in X+Y by simply ignoring the carry bit. However, a modular reduction is much more expensive at X∗Y. Moreover, for an RNS circuit to perform the modular reduction, it has to know the modulus $m_{i}$. This creates yet another vulnerability—wherein the RNS circuit has to protect $m_{i}$. For an adversary model, where $m_{i}$ must be kept secret at an encoder/client cloud node, it would be beneficial not to have to perform modular reduction with respect to $m_{i}$ on-chip. The modular reduction can be delayed significantly through the use of Montgomery multiplication [28]. In summary, Montgomery reduction is performed in a field that is a power of two so that a processor can perform it efficiently. This defers actual $m_{i}$ modular reduction to the circuit boundaries. We evaluated Montgomery reduction-based RNS circuits for machine learning-based secret leakage and for correlation power attacks (CPA) effectiveness. We have implemented a Montgomery reduction scheme on the 3-bit residue shares with the auxiliary modulus $2^{3}$. The architecture was designed based on the idea proposed in [29], and the required area is 253 GE (gate-equivalent). We have measured the peak current, and the power consumption for 25,000 randomly generated inputs and studied the SCA metrics.

In Montgomery multiplication, the reduction modulus is required to be an integral power of two, which forces the modulus $m_{i}$ to an odd value, given that the reduction modulus needs to be co-prime with the original modulus. Montgomery reduction reduces the available modulus set ($m_{i}$) for a given *l*-bit representation by eliminating even moduli. We have constructed a hardware structure for modular reduction called Arithmetic modular reduction in order to maximize this set. In Section 3, we stated that for practical hardware circuits, the residue size is limited to small values, such as 3 or 5. This allows us to compute the canonical form for modular reduction function on residue size of three. The circuit implementation was done using FreePDK 45 nm standard cell library whose area is 582 GE. We performed circuit simulations to capture the peak current values for Montgomery reduction and Arithmetic reduction schemes.

We compare Arithmetic modular reduction schemes against Montgomery reduction schemes for power side-channel attack resistance [25] in Table 6. The KL divergence value for Montgomery reduction is 0.0204, which corresponds to 90% failure probability. The SCA metric with KL divergence for Arithmetic modular reduction is 0.0024, and the corresponding failure probability is close to 99%. Note that the power consumption of the Arithmetic reduction scheme is higher than that of the Montgomery reduction schemes, with a more uniform peak current profile. We also applied ML-classifiers to determine the secret bit. The success rates of various classifiers are given in Table 7. The success rates of Montgomery reduction and Arithmetic modular reduction are around 35%, showing protection compared to the random guess success rate of 50%. The result clearly shows that the adversary does not have any significant advantage over a random guess of the secret values.

**Figure 9:**
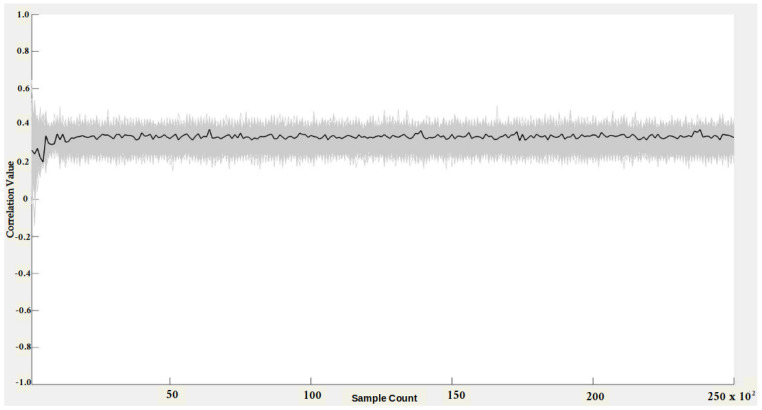
CPA attack on Montgomery multiplier.

We have also evaluated the side-channel security for both Montgomery reduction and Arithmetic modular reduction to determine the minimum number of samples to reveal the secret using the CPA tool. The objective of a CPA adversary is to infer the secret input value by correlating the measured power consumption with the power model derived for the target implementation. We generated 25,000 random values for control inputs. Corresponding residue shares using RNS encoding with random value $r_{x}$ = 3 and modulus $m_{i}$ = 7 were then created. The secret input *y*=1 was also encoded with the random value $r_{y}$ = 5 using RNS encoder. The residue share values were input to the Montgomery reduction block, whose power was captured. The hypothetical power model was derived by targeting the output of the Montgomery reduction using a hamming distance power model. The hypothetical matrix was generated for unknown space of size $2^{7}$, i.e., secret input (1-bit) + random value $r_{x}$ (3-bit) + random value $r_{y}$ (3-bit) = total (7-bit). With the modulus value, the unknown search space size increases to $2^{10}$, which we are unable to process with our computational resources. We correlated the measured power consumption with the hypothetical power model, and the results are reported in Figure 9.

**Figure 10:**
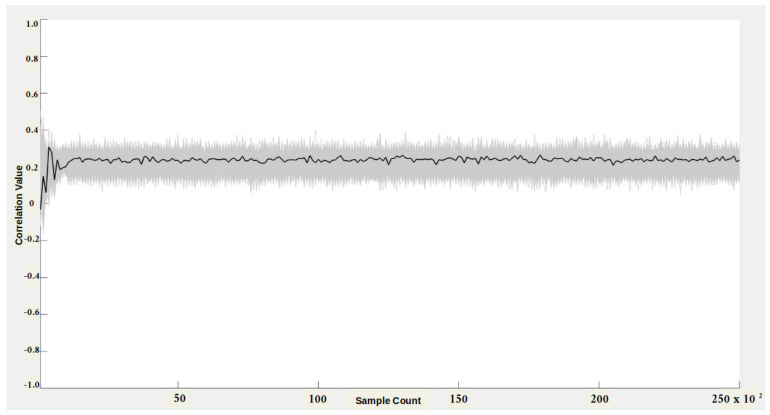
CPA attack on Arithmetic multiplier.

In Figure 9, the black represents the correct key hypotheses, and the wrong key guess hypotheses are highlighted in gray. The wrong keys envelop the correct key hypothesis, and also, there is no distinct peak in the correlation. Hence, the adversary does not have any advantage in distinguishing the correct secret value from the raw search space. We also believe that adding the modulus value to the search space will increase the complexity for the adversary. We conducted a similar experiment on an Arithmetic-style implementation, and the results are given in Figure 10. We used the same set of random values for both experiments. The hypotheses results remained the same for the Arthimetic multiplier; the secret value has low correlation values compared to wrong key hypotheses. Thus, the CPA adversary failed to recover secret key values from Montgomery reduction and Arithmetic reduction circuits.

### 5.2. FPGA Evaluation

**Figure 11:**
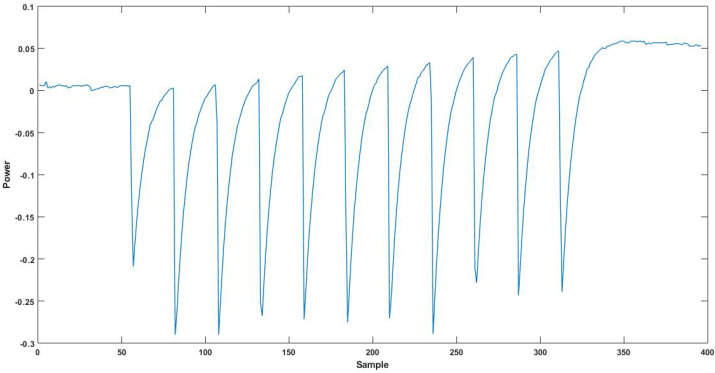
Power trace of AES RNS circuit.

To evaluate the security of our scheme in a physical platform, we implemented AES encryption on the Sakura-G board using Xilinx ISE 14.6. The board consists of two spartan-6 FPGAs: the XC6SLX9 device contains a communication protocol to send/receive data between analysis PC and victim FPGA (XC6SLX75). The board interface was based on openADC to capture the power trace using chipwhisperer software [30]. We implemented both a base design with no protections and the RNS secure design of AES encryption in the victim FPGA. The unprotected AES was implemented in a round-based architecture, which takes 128-bit plaintext and 128-bit key as input and generates the 128-bit ciphertext. The state array updates intermediate around results with a 128-bit register. A key choice in the RNS circuit design is modulus size, which effects the RNS circuit design complexity and security. In this design, we picked 3-bit moduli. With this design choice, we needed three RNS shares. RNS encoding converts each plaintext bit into three 3-bit RNS shares using the modulo values 3, 4, and 5. The modulo values were chosen such that they were co-prime to each other. The RNS circuit, for each share/lane, was implemented on the victim FPGA separately. Resource utilization is given in Table 8. The RNS-protected AES circuit requires 6063 FPGA slices, which is six times the slice needs of unprotected implementation. The RNS-protected implementation takes RNS shares as input and computes the output represented in RNS shares. The key expansion and AES core round functions were constructed using RNS logic with a 384-bit state register($S_{i}$) to store the intermediate values, where *i* represents the round number. The configurations and experimental setup details are listed in Table 9. We measured 100,000 power traces of the victim FPGA during the ten rounds of the secure RNS AES circuit as the voltage drop across 1Ω resistor, as shown in Figure 11.

**Figure 12:**
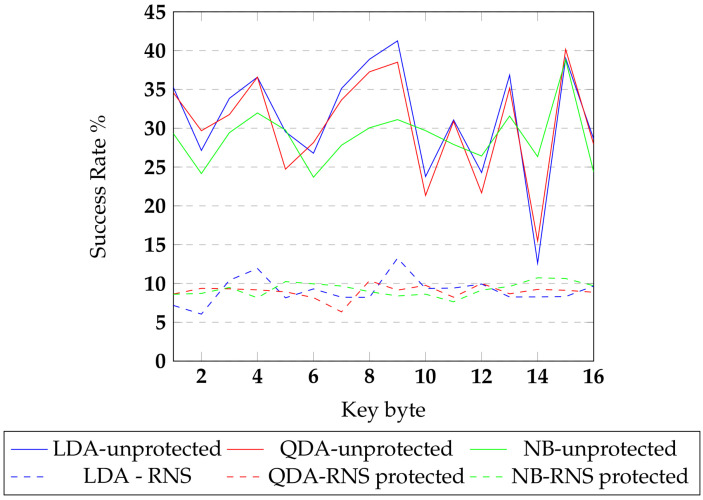
Classifier results on secure implementation.

Evaluation of the RNS-protected AES implementation using machine-learning classifiers was based on features extracted from the power traces. The feature vector was created with peak power consumption values and the Hamming distance value of the state registers. The state register update caused significant power consumption synchronized with clock cycles. The Hamming distance between the RNS output $S_{10}$ and $S_{9}$ round values was calculated and introduced into feature arrays to perform byte-level classification. The feature vector array was labeled with corresponding RNS key byte values for key expansion of the tenth round. The machine learning classifiers LDA, QDA, and naive Bayes, were used to predict the key values from the feature vector array. The success rates of key byte prediction for various classifiers and implementations are shown in Figure 12. The continuous line represents the success rate values for the unprotected AES, and the dashed lines represent the success rate values of the RNS-protected implementation. From the results, it is observed that the protected implementation had a success rate that is 79.66% lower than the success rate of the base implementation. In RNS circuits, the input encoding and output decoding functions are off-chip computations. This makes it difficult for the traditional CPA attack to reveal the binary secrets.

## 6. Conclusions

IoT nodes in a cyber-physical system are attractive targets for physical side-channel attacks. The physical side-channel attacks benefit from the physical possession or being in the vicinity of the device. This paper has presented a novel logic design style based on residue number systems that offer increased resistance to power side-channel attacks.

We have developed new secure logic based on secret sharing and residue number system. We illustrated the transformation of a boolean function representation into residue operations, such as modular multiplication and modular addition. Several variants of secure RNS logic family based on the encoder design and number of independent random variables are presented. We develop the resilience characteristics of the RNS secure circuits against a power analysis attack. KL divergence captures the statistical differentiability of the power trace distribution for various secret values. A low KL divergence value signifies that the differentiability is very low making the circuit side-channel leakage resistant. Our results show KL divergence value of 0.1165 for 3-bit residue designs. For residue representation with small modulus values, an adversary has significant cryptanalytic capability to model the relationship between a primary input bit and its residues. Several variants of the secure RNS logic family varying in the encoder design and number of independent random variables were presented. The enhancement techniques, such as random renewal and hybrid scheme, restore the switching uniformity in the RNS residues and increase the entropy of the moduli’s space.

The resistance of the RNS secure logic family was studied for a boolean AND gate. It was quantified using normalized variance and KL divergence as SCA metrics. We also studied the success rates of common machine learning classifiers such as QDA, LDA, and naive Bayes. The SPICE simulations for standard RNS circuits resulted in a KL divergence value of 4.539, whereas the random renewal scheme and hybrid scheme with *t*-private logic exhibit much reduced KL divergence of 1.8409 and 0.7312, respectively. This attests to the increased side-channel resistance of random renewal and hybrid schemes. The machine learning success rate based SCA metric shows that these enhancements improve the targeted design resistance.

The RNS secure logic can be supported with both public and private moduli. We incorporated Montgomery reduction-based multiplication and its variant—Arithmetic reduction—to enable private moduli. The KL divergence for Montgomery and Arithmetic reductions were 0.0204 and 0.0024, respectively. This paper also presents a protected AES implementation using RNS secure logic on an FPGA platform. The side-channel security was evaluated using ML classifier success rates on the real signals collected from this FPGA. The protected implementation resulted in a 79.66% lower success rate (higher resistance) compared to an unprotected AES circuit. These results collectively show that the RNS logic exhibits high resistance to power analysis attacks.

## Figures and Tables

**Table 1:** Sample residue computation for l=2.

*x*	*r*	$X_{m_{1}}$	$X_{m_{2}}$
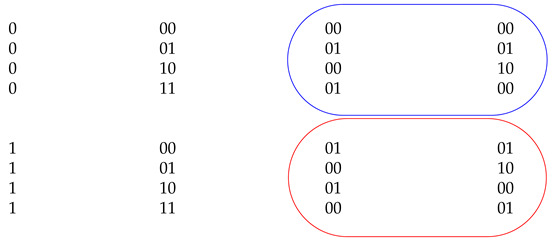			

**Table 2:** Results of symmetry and ML classification for residue shares.

Classifier	Success Rate		
	*l* = 3	*l* = 4	*l* = 5
LDA	50.05%	51.39%	50.57%
QDA	52.08%	50.89%	50.68%
Naives Bayes	62.50%	47.96%	47.92%
Symmetry	0.80	0.91	0.99
KL divergence($DL_{max}$)	0.1165	0.0227	0.00634

**Table 3:** SCA metrics for various RNS schemes with a single random value.

Transition	RNS Secure Circuit		Random Renewal Scheme		Random Renewal with *t*-Private	
	Power (μW)	Peak Current (mA)	Power (μW)	Peak Current (mA)	Power (μW)	Peak Current (mA)
0 → 0	103.06	1.04	799.81	2.616	1112.56	4.505
0 → 1	101.49	0.88	798.86	2.595	1114.35	4.511
1 → 0	102.25	0.65	799.30	2.610	1107.93	4.507
1 → 1	103.65	0.65	798.21	2.586	1109.50	4.508
Average (μ)	102.618	0.862	799.05	2.602	1111.12	4.508
Standard Deviation (σ)	0.945	0.158	0.0058	0.0120	0.00026	0.0022
Coefficient of Variation ($\frac{\sigma }{\mu }$)	0.0092	0.1835	0.00007	0.0004	0.000023	0.00049
KL Divergence ($DL_{max}$)	-	4.539	-	1.8409	-	0.7312

**Table 4:** SCA Metrics for various RNS schemes with separate random values.

Transition	RNS Secure Circuit		Random Renewal Scheme		Random Renewal with *t*-Private	
	Power (μW)	Peak Current (mA)	Power (μW)	Peak Current (mA)	Power (μW)	Peak Current (mA)
0 → 0	102.78	0.92	799.80	2.620	1113.70	4.517
0 → 1	102.00	1.05	798.86	2.618	1115.00	4.525
1 → 0	102.07	1.03	799.29	2.616	1109.80	4.515
1 → 1	104.90	0.77	798.21	2.617	1110.70	4.516
Average (μ)	102.93	0.947	799.04	2.618	1112.30	4.518
Standard Deviation (σ)	1.351	0.128	0.0058	0.0015	0.0002	0.00401
Coefficient of Variation ($\frac{\sigma }{\mu }$)	0.0131	0.1348	0.00007	0.00057	0.000017	0.00088
KL Divergence ($DL_{max}$)	-	1.212	-	0.1620	-	0.0688

**Table 5:** Success rate on *t*-private, base RNS, random renewal, random renewal with *t*-private with a single shared random variable.

Classifier	*t*-Private	RNS Secure Circuit	Random Renewal Scheme	Random Renewal with *t*-Private
LDA	36.9%	25.28%	25.05%	30.80%
QDA	31.4%	25.55%	25.57%	35.71%
Naives Bayes	40.3%	27.64%	24.74%	23.49%

**Table 6:** SCA metrics for modular multiplication.

Transition	Montgomery Modular Multiplication		Arithmetic Modular Multiplication	
	Power (μW)	Peak Current (mA)	Power (μW)	Peak Current (mA)
0 → 0	1616.91	4.9815	2224.20	6.8296
0 → 1	1613.20	4.9711	2224.10	6.8305
1 → 0	1614.90	4.9759	2224.50	6.8315
1 → 1	1611.21	4.9642	2224.50	6.8314
Average (μ)	1614.06	4.9732	2224.33	6.8307
Standard Deviation (σ)	2.10298	0.007359	0.178536	0.000769
Coefficient of Variation ($\frac{\sigma }{\mu }$)	0.00130	0.00147	0.00008023	0.00112
KL divergence ($DL_{max}$)	-	0.0204	-	0.0024

**Table 7:** Classifier output for modular multiplication.

Classifier	Montgomery	Arithmetic
LDA	36.64%	33.72%
QDA	37.04%	35.77%
Naives Bayes	19.48%	22.78%

**Table 8:** Hardware resource utilization of AES.

Implementation	Slice Registers	Slice LUTs	Slice Occupied
AES encryption	1002	3208	998
AES—RNS circuit Mod3	1437	7089	1971
AES—RNS circuit Mod4	1437	7158	1994
AES—RNS circuit Mod5	1437	7913	2098
Total Resources (Mod3 + Mod4 + Mod5)	4311	22,160	6063

**Table 9:** Experimental setup parameters.

Parameter	Length/Size
AES plaintext size	128 bit
AES secret key size	128 bit
AES plaintext residue share size	384 bit
AES secret key residue share size	384 bit
size of residue share per bit (*l*)	3 bit
modulus values	3 bit
data recorded	100,000
training dataset	80,000
test dataset	20,000

## Data Availability

Not applicable.

## Abbreviations

The following abbreviations are used in this manuscript:

RNS	Residue Number System
ML	Machine Learning
AES	Advanced Encryption Standard
SCA	Side-Channel Attack
DPA	Differential Power Analysis
HO-SCA	Higher-Order Side-Channel Attack
SABL	Sense Amplifier Based Logic
WDDL	Wave Dynamic Differential Logic
LDA	Linear Discriminant Analysis
QDA	Quadratic Discriminant Analysis
NB	Navie Bayes
KL divergence	Kullback–Leibler divergence
CRT	Chinese Remainder Theorem
CPA	Correlation Power Analysis
FPGA	Field Programmable Gate Array
IoT	Internet of Things
GE	Gate Equivalence
